# Body Schema Illusions: A Study of the Link between the Rubber Hand and Kinesthetic Mirror Illusions through Individual Differences

**DOI:** 10.1155/2017/6937328

**Published:** 2017-10-23

**Authors:** Morgane Metral, Corentin Gonthier, Marion Luyat, Michel Guerraz

**Affiliations:** ^1^LPNC (UMR CNRS 5105), Université Savoie Mont Blanc, Chambéry, France; ^2^LP3C (EA 1285), Université Rennes 2, Rennes, France; ^3^PSITEC (EA 4072), Université Lille 3, Lille, France

## Abstract

**Background:**

The well-known rubber hand paradigm induces an illusion by having participants feel the touch applied to a fake hand. In parallel, the kinesthetic mirror illusion elicits illusions of movement by moving the reflection of a participant's arm. Experimental manipulation of sensory inputs leads to emergence of these multisensory illusions. There are strong conceptual similarities between these two illusions, suggesting that they rely on the same neurophysiological mechanisms, but this relationship has never been investigated. Studies indicate that participants differ in their sensitivity to these illusions, which provides a possibility for studying the relationship between these two illusions.

**Method:**

We tested 36 healthy participants to confirm that there exist reliable individual differences in sensitivity to the two illusions and that participants sensitive to one illusion are also sensitive to the other.

**Results:**

The results revealed that illusion sensitivity was very stable across trials and that individual differences in sensitivity to the kinesthetic mirror illusion were highly related to individual differences in sensitivity to the rubber hand illusion.

**Conclusions:**

Overall, these results support the idea that these two illusions may be both linked to a transitory modification of body schema, wherein the most sensitive people have the most malleable body schema.

## 1. Background

Optical illusions may be the first example of illusions that spring to mind. Yet other types of illusions are at least as impressive. Take, for instance, bodily illusions based on manipulations of the body schema. The body schema is an internal and dynamic representation of the body, of the relative positions of body parts, and of their relative metrics [1–4]. It is essentially a sensorimotor representation, built and updated at an unconscious level on the basis of visual, tactile and proprioceptive information [4]. This representation can be experimentally tricked by manipulating multisensory integration rules. The resolution of conflict between multiple sensory signals (of which some are congruent and others incongruent) can lead, even in healthy subjects, to a wrong representation of some body parts or even the entire body. There are several types of body schema illusions, such as the marble hand illusion [5], the invisible body illusion [6], the rubber hand illusion [7], and the kinesthetic mirror illusion [8]. In the present study, we focused on the rubber hand illusion and the kinesthetic mirror illusion, to determine whether these two tasks are linked.

The well-known rubber hand illusion is a prominent example of body schema manipulation (for the seminal study, see [7]). In this paradigm, the sensation that a fake rubber hand belongs to one's own body is induced by repeated tactile stimulation of one of the participant's hands, hidden from view, and concomitant visual stimulation of a fake rubber hand placed in front of the participant. In this particular illusion, there is a temporal and spatial congruence between visual inputs (seeing a rubber hand being stroked) and tactile inputs (simultaneously feeling one's own hand being stroked). Even though these two sources of information are incongruent with proprioceptive information from the real hidden hand, the temporal and spatial match between visual input (seeing a rubber hand being stroked) and tactile input (at the same time and at the same location feeling the own hand being stroked) is sufficient for the brain to integrate the two events into a single event. Participants have thus the illusory feeling that they perceive tactile stimuli on the rubber hand and that their own hand tends to acquire the position of the fake one [7, 9–11]. As soon as temporal congruence between visual and tactile inputs is broken, this illusory feeling decreases.

Other examples of illusions affecting the body schema can be obtained using mirrors. Mirror illusions were first used as a tool to reduce phantom limb pain after amputation [12]. The authors placed a mirror in front of the patient along the midsagittal axis of the body. The affected limb was hidden behind the mirror, while the patient viewed their intact limb reflected in the mirror. The mirror reflection mimicked the visual appearance of the amputated limb; this restored congruence between visual and proprioceptive inputs and evoked the feeling that the amputated limb had been “resurrected” in some patients [13].

In healthy subjects, the same mirror configuration can also be used to induce multiple motor and perceptual responses on the arm hidden behind the mirror [14]. For example, the mirror can lead to directional biases in reaching movements on the contralateral hand hidden behind the mirror [15, 16] and could also enhance bimanual coordination [17, 18]. Moreover, viewing the reflection of one's arm being passively moved induces consistent, vivid kinesthetic illusions of movement on the static arm hidden behind the mirror; this effect has been called the kinesthetic mirror illusion [8, 19, 20].

Although the rubber hand illusion and the kinesthetic mirror illusion use different materials, they seem to work in very similar ways. It is well established that both illusions can be explained as resulting of the integration of conflicting visual and somatosensory inputs [14, 21]. In the case of the rubber hand illusion, many authors agree that the bias in sensory referral is associated with a modulation of the body schema (see Tsakiris, [22]). Likewise, mirror illusions used for the treatment of phantom pain have been attributed to modulation of the sensory representation of the hidden limb by visual mirror feedback of the limb facing the mirror [21] and the kinesthetic mirror illusion is presumably due to the same mechanism. In support of this idea, the strength of both illusions is reduced by anatomical and postural discrepancies between the visible, fake, stimulated body part (rubber hand or reflected arm) and the actual body part occluded from view [20, 23, 24]. This similarity suggests that the two illusions could be manifestations of the same phenomenon of body schema modulation. Past studies have often drawn a parallel between the two illusions [14, 21, 25]. However, to our knowledge, the relationship between the two illusions has not yet been tested.

One possibility of investigating the relationship between these two illusions would be to study individual differences. The body schema is not a universal, innate representation; instead, it is subject to variability. For example, development of the body schema depends on the functioning multisensory integration. Multisensory foundations of the body schema only reach an adult state at 10 to 11 years of age, which indicates intraindividual variability [26].

Illusory feelings affecting the body schema are consistently variable and depend on the participant; in particular there are individual differences in sensitivity to the kinesthetic mirror illusion and the rubber hand illusion. For instance, in the context of experiments performed in our own lab (e.g., [19, 20, 27]), we estimate that about one-fifth of participants are not sensitive to the kinesthetic mirror illusion. Indeed, 28% of participants did not respond to the illusion in [19], 18% in [20], and 16% in [27]. In the rubber hand illusion, interindividual differences upon multisensory temporal binding window—the epoch of time within which stimuli from different modalities is likely to be integrated and perceptually bound—explained sensitivity toward the illusion of ownership [28]. Another example of these individual differences is found in the rubber hand illusion, between healthy participants and eating disorders patients. People who are suffering from eating disorder are more likely to be sensitive to the rubber hand illusion [10, 29, 30]. This is also true in patients with a former eating disorder who have recovered [30]. Participants especially responsive to the rubber hand illusion seem to more readily accept inaccurate bodily information as valid, as if their body schemas were especially malleable [10]. In other words, it seems to be the case that people more sensitive to the rubber hand illusion assign more weight to external visual input (rubber hand), relative to internal bodily information (real hand). Because patients who have recovered from an eating disorder still demonstrate particular sensitivity, Costantini et al. [28] have hypothesized that this sensitivity may exist prior to the development of an eating disorder. In other words, people who are more likely to develop an eating disorder would have a more malleable representation of their body, which would make them more sensitive to the rubber hand illusion, and presumably to other body schema illusions such as the kinesthetic mirror illusion.

In summary, both the rubber hand illusion and the kinesthetic mirror illusion conceptually appear to rely on the same mechanism of body schema manipulation. There are also individual differences in the sensitivity to these illusions, which have been attributed to individual differences in the malleability of the body schema. These individual differences provide an adequate basis to determine whether the rubber hand illusion and the kinesthetic mirror illusion do rely on the same mechanism: if malleability of the body schema is the source of the illusion in both paradigms, participants who are more sensitive to the rubber hand illusion should also be more sensitive to the kinesthetic mirror illusion.

The main purpose of the present study was to test this hypothesis. A sample of nonclinical participants completed both the rubber hand and the kinesthetic mirror illusion paradigms, and their sensitivity to both illusions was assessed. To ensure that differences between participants reflected stable individual differences in illusion sensitivity, rather than random variation, illusion strength was assessed at two different time points based on three different measures for each illusion. Participants also completed control conditions for each illusion to ensure that illusory responses were specific to body schema manipulation. Lastly, the convergence between sensitivity to the rubber hand and kinesthetic mirror illusions was assessed. The two measures were expected to correlate, compatible with the hypothesis of a common mechanism of body schema manipulation.

Additionally, some studies have shown a relationship between sensitivity to body schema illusions and eating disorders [10, 29, 30], raising the possibility that individual differences in malleability of the body schema are predictive of the risk of developing an eating disorder. Of secondary interest, nonclinical participants in our study completed questionnaires related to eating disorders to determine whether individual differences in sensitivity to the two illusions would be associated with traits related to eating disorders, as suggested by the literature [10, 29, 30].

## 2. Method

### 2.1. Participants

Thirty-six undergraduates in psychology (mean age = 21.30 years, SD = 6.36) completed the experiment. Only women were included in the sample to avoid gender effects on body perception (previous studies have shown that women are more likely to develop body dissatisfaction and eating disorders [31, 32]). All but two participants were right-handed (as determined using the Edinburgh Inventory Test [33]). None of the 36 volunteers had a history of visual, proprioceptive or neuromuscular disease, and none had a history of eating disorders as declared in a self-report questionnaire. Their average body mass index (BMI, i.e., mass/height^2^) was 21.45 (SD = 2.87).

### 2.2. General Experimental Procedure

All participants provided written informed consent prior to completing the study. The experiment was performed in accordance with the declaration of Helsinki. Each participant was tested individually in a quiet room. They completed both the kinesthetic mirror illusion task and the rubber hand illusion task; details are provided in the next sections. The participant completed two sessions of the kinesthetic mirror illusion task (KMI-1 and KMI-2) and two sessions of the rubber hand illusion task (RHI-1 and RHI-2) to confirm that there were stable individual differences in time. The two illusions were interleaved and order was counterbalanced across participants, so that half of the participants completed KMI-1, RHI-1, KMI-2, and RHI-2, and the other half completed RHI-1, KMI-1, RHI-2, and KMI-2. The participant performed control conditions for both illusion tasks to confirm that responses of feeling the illusions were not due to social desirability. Each session of the kinesthetic mirror illusion task included four trials in the illusion condition and two trials in control conditions; each session of the rubber hand illusion task included two trials in the illusion condition and two trials in a control condition. The number of trials was doubled in the illusion condition of the kinesthetic mirror illusion, given the short duration of trials in this illusion (18 sec) when compared to the rubber hand illusion (90 sec). It must be mentioned that results were comparable when analyzing only the first two trials to equalize the number of trials across all conditions. Illusion and control conditions were performed in random order. At the end of the procedure, the participant completed two questionnaires related to eating disorders and their height and weight were measured to allow for computation of their BMI.

### 2.3. Eating Disorders Questionnaires

The Body Shape Questionnaire (BSQ) is a one-dimensional, 34-items self-report questionnaire that assesses the frequency of concerns about body shape over the preceding four weeks [34]. The answers on each item are rated on a Likert scale from 1 (concern* not present*) to 6 (concern* always present*), and a total score is computed as the sum of all answers. Possible scores range from 34 to 204, with higher scores indicating more concern about body shape. We used the French validation of the BSQ [35].

The Eating Disorders Inventory-2 (EDI-II) is a self-report questionnaire with 91 items and 11 subscales measuring symptoms and psychological traits commonly associated with eating disorders [36]. Three subscales measure eating-related symptoms:* drive for thinness*,* bulimia*, and* body dissatisfaction*. The other eight subscales measure psychological traits characteristic of patients with eating disorders (e.g.,* interpersonal distrust*,* fear of maturity*, and* perfectionism*). Responses are rated on a Likert scale from 1 (*never*) to 6 (*always*). A total score is then computed as the sum of all answers, with higher scores indicating more pathological traits. We used the French validation of the questionnaire [37].

### 2.4. Kinesthetic Mirror Illusion Task

#### 2.4.1. Materials

**Figure 1:**
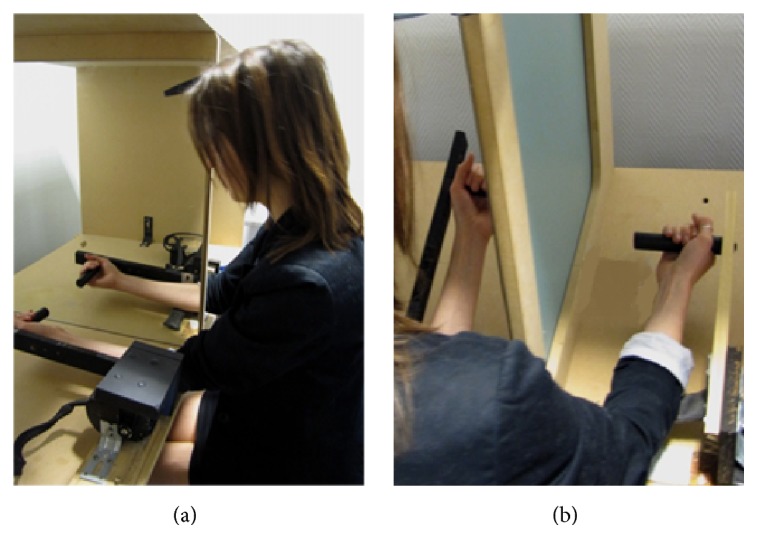
Illustration of the experimental setup for the kinesthetic mirror illusion task. The participant sat at a table and faced a box compartmentalized by a vertical mirror. (a) The participant's left arm was supported by a motorized manipulandum that could flex the arm. Visible to the participant, the vertical mirror reflected the image of their left arm. (b) Invisible to the participant, their right arm was supported by a static manipulandum and held at a 15° angle.

The participant sat in front of a large, custom-built box (see Figure 1 for an illustration). A mirror (measuring 65 cm by 65 cm) was positioned vertically in the middle of the box and was oriented parallel to the participant's mid-sagittal plane, with the reflective surface facing the participant's left side. The participant's forearms were positioned on either side of the mirror and were held by two manipulanda devices (wooden arms on which subjects placed their forearms and hands). The distance between the manipulanda and the mirror was adjusted so that the mirror image of the left arm mimicked the position of the right arm, which created a “false” right arm from the point of view of the participant. The right manipulandum was fixed, whereas the left manipulandum was motorized (with a low-noise direct current motor) and could be rotated via a remote controller to flex the participant's left elbow joint.

#### 2.4.2. Procedure

The participant was positioned facing the mirror, with their right arm hidden behind the mirror and only the left arm facing the mirror being visible (see Figure 1). They were instructed to always keep looking at the reflection of their left arm in the mirror. The participant's left forearm was adjusted on the manipulandum so that the axis of motorized rotation coincided with the elbow joint. In the starting position, both manipulanda were positioned at 15 degrees above the horizontal in the sagittal plane (see Figure 1). The right arm always remained stationary. The participant first completed a few practice trials with a passive displacement of the left forearm until they were sufficiently familiar with the material and instructions.

In the illusion condition, the left forearm was passively flexed at a constant velocity of 4° per second for 18 seconds, from its starting position at 15° to a final of position at 85° above the horizontal. The participant was required not to resist to this passive displacement. Each experimental trial started when the manipulandum initiated its movement. In this condition, the participant was expected to experience the kinesthetic mirror illusion (i.e., the sensation that their stationary right arm was moving along with the reflection of their left arm). In addition to this illusion condition, the participant completed two control conditions: a control condition without displacement of the left arm, where the participant saw the reflection of their static left arm in the mirror, and a control condition without a mirror, where the participant's left arm was flexed but the mirror was replaced by an opaque board. No illusion was expected in either control condition.

#### 2.4.3. Data Collection

Three measures were collected in each trial and averaged over all trials in a session (KMI-1 and KMI-2 sessions): proprioceptive drift, onset latency, and subjective speed of the illusion. (1) Before and after each trial, the participant was required to estimate the angular position of their right hidden forearm relative to the sagittal axis (in degrees, from 0° to 90°). This measure reflected proprioceptive drift. (2) During each trial, the participant was required to immediately report to the experimenter if she felt that her hidden, stationary right arm started moving. The delay between the beginning of the trial and the moment the participant felt the illusion (in seconds) was recorded by the experimenter using a stop watch. This delay reflected onset latency of the kinesthetic illusion. If the participant did not report feeling the illusion at any point, onset latency was recorded as the total duration of the trial (18 seconds). (3) At the end of each trial, the participant was required to rate the perceived speed of the displacement of their right forearm during the trial, on a scale ranging from 0 to 20 with steps of 1. The answer 0 corresponded to feeling no displacement at all, 10 corresponded to feeling a displacement with velocity equal to that of the passively moved left forearm, and 20 corresponded to feeling a displacement with velocity equal to twice that of the passively moved left forearm. This measure reflected subjective speed of the kinesthetic illusion (for a similar procedure, see [8]).

To summarize the illusion strength with a single value reflecting individual differences as precisely as possible, a composite illusion strength index was also computed by aggregating the values obtained for proprioceptive drift, onset latency and subjective speed. The three values were first standardized (transformed into *z*-scores). The latency *z*-score was reversed to yield a measure on the same scale as the two other indices (with larger values representing a stronger illusion). The three *z*-scores were then averaged to yield the composite index of the kinesthetic mirror illusion.

### 2.5. Rubber Hand Illusion Task

#### 2.5.1. Materials

**Figure 2:**
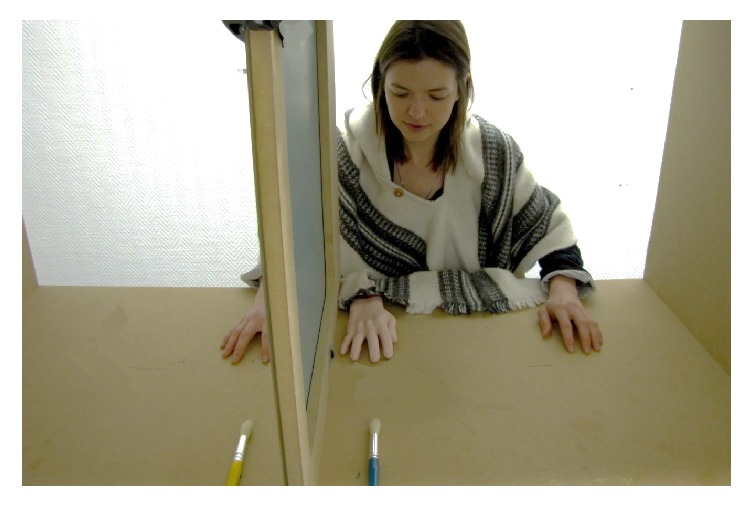
Illustration of the experimental setup for the rubber hand illusion task. The participant sat at a table and faced a box compartmentalized by a vertical opaque board. The participant's right hand was occluded from view by the opaque board; the rubber hand was placed in front of the participant.

The participant sat in front of a custom-built box (see Figure 2 for an illustration), with an opaque board positioned vertically in the middle. The participant's forearms were placed on either side of the opaque board and laid directly on the table. The participant could not see their right hand, hidden behind the opaque board. A life-size rubber model of a right hand and wrist was placed on the table directly in front of the participant.

#### 2.5.2. Procedure

The participant was seated in front of the rubber hand setup, with their forearms on the table with the palms down, and with the index fingers on two marked positions. The index of the rubber hand and the index of the participant's right hand were both positioned at a distance of 10 cm from the opaque board (i.e., they were separated by a distance of 20 cm). They were instructed to always keep looking at the rubber hand.

Each trial lasted 90 seconds. In the illusion condition, the experimenter synchronously stroked the rubber hand and the participant's right hand with two soft brushes. The strokes were performed at the same time, at the same frequency, and on the same location for both the fake hand and the real hand. Strokes always went from the top of the hand, just above the knuckle, toward the fingertip, with a constant frequency of approximately one stroke per two seconds. In this condition, the participant was expected to experience the rubber hand illusion (i.e., feel the touch applied to the fake hand, as if it was incorporated in their body schema). In the control condition, the strokes were asynchronous: the fake hand and the real hand were touched at different times in different locations. No illusion was expected in this condition.

#### 2.5.3. Data Collection

As in the kinesthetic mirror illusion, three measures were collected in each trial and averaged over all trials in a session (RHI-1 and RHI-2 sessions): proprioceptive drift, onset latency, and subjective intensity of the illusion. (1) Before and after each trial, the participant was required to estimate the location of their hidden right index finger (for a similar procedure, see, e.g., [10]). For this particular measure, an opaque tissue was placed upon the experimental setup to prevent the participant from seeing their own hands and the rubber hand inside the frame. The experimenter moved a vertical metal bar (length: 45 cm) alongside the top of the experimental setup in such a way that the participant could see it. The participant was instructed to say “stop” as soon as the location of the vertical bar matched the perceived location of the middle of their hidden right index finger. Proprioceptive drift was computed as the difference between the estimated location of the index finger after and before the trial (in cm). Higher distance indicating more proprioceptive drift toward the rubber hand. (2) During each trial, the participant was required to immediately report to the experimenter if they felt the rubber hand illusion. Criteria for feeling the rubber hand illusion were defined based on the first three items of the* embodiment questionnaire* [7, 38]: (i) feeling the touch of the paintbrush in the location where they saw the rubber hand touched, (ii) feeling a touch as if it was caused by the paintbrush touching the rubber hand, or (iii) feeling as if the rubber hand was their own hand. It comprises 10 items rated on a 10-point Likert scale ranging from 1 (strongly disagree) to 10 (strongly agree). The first three items reflect illusory ownership of the rubber hand, and the seven others are control items (e.g., “It felt as if my real hand were turning rubbery”). The delay between the beginning of the trial and the moment the participant felt the illusion (in seconds) was recorded by the experimenter using a stop watch. This delay reflected onset latency of the rubber hand illusion. If the participant did not report feeling the illusion at any point, onset latency was recorded as the total duration of the trial (90 seconds). (3) After each trial, the participant filled out the 10-item embodiment questionnaire (as defined by Kammers and colleagues [38], adapted from the original version [7]). The first three statements have been shown to specifically measure experience of ownership over the rubber hand. Subjective intensity of the rubber hand illusion was also computed as the average rating on the first three items of the questionnaire. As in the kinesthetic mirror illusion, a composite illusion strength index was also computed by aggregating the values obtained for proprioceptive drift, onset latency, and subjective intensity.

## 3. Results

The raw data are available in Supplementary Material available online at https://doi.org/10.1155/2017/6937328. Descriptive statistics are displayed in Table 1. Because the distribution of scores was markedly nonnormal for several variables, all analyses were performed using nonparametric statistics. To ensure the robustness of our data, additional skipped nonparametric statistics [39] were performed. Given that they provided similar results, they were not further presented.

### 3.1. Comparison between Illusion and Control Conditions

For the kinesthetic mirror illusion task, none of the 36 participants reported any kinesthetic illusion in the two control conditions (conditions without arm displacement and without mirror vision), confirming that the illusion only appeared when looking at the reflection of the left arm being passively moved.

For the rubber hand illusion, the difference between illusion strength measures in the experimental condition (synchronous strokes) and the control condition (asynchronous strokes) was tested using Wilcoxon's signed-rank test. Illusion strength was significantly greater in the illusion condition for proprioceptive drift (*Z* = 4.23, *p* < .001), for onset latency (*Z* = 4.78, *p* < .001), and for subjective intensity (*Z* = 5.09, *p* < .001), confirming the presence of the rubber hand illusion in the illusion condition.

### 3.2. Reliability of Illusion Strength Measures

Test-retest stability of illusion strength measures was assessed by computing their correlation over the two sessions of trials using Spearman's rho. For the kinesthetic mirror illusion, the analysis carried out high positive and significant correlations between the first session and the second session for onset latency (*ρ* = .84, *p* < .001), subjective speed (*ρ* = .87, *p* < .001), proprioceptive drift (*ρ* = .97, *p* < .001), and the composite illusion strength index (*ρ* = .91, *p* < .001).

For the rubber hand illusion, the correlation between first session and second session was also very high for onset latency (*ρ* = .96, *p* < .001), subjective rating (*ρ* = .98, *p* < .001), and the composite illusion strength index (*ρ* = .95, *p* < .001) and still acceptable for proprioceptive drift (*ρ* = .72, *p* < .001). In short, participants demonstrated stable individual differences in their sensitivity to the kinesthetic mirror and to the rubber hand illusions. Given the very high test-retest reliability of illusion strength indices, subsequent analyses were performed by averaging scores collected in the two sessions of trials.

### 3.3. Correlations between Illusion Strength Measures

The matrix of all bivariate correlations between illusion strength measures is displayed in Table 2. Overall, different illusion strength measures within the same illusion were significantly correlated, except for the rubber hand illusion where the proprioceptive drift index did not correlate with either onset latency or subjective rating. These correlations confirm that participants demonstrated consistent individual differences in illusion sensitivity across measurement types.

**Figure 3:**
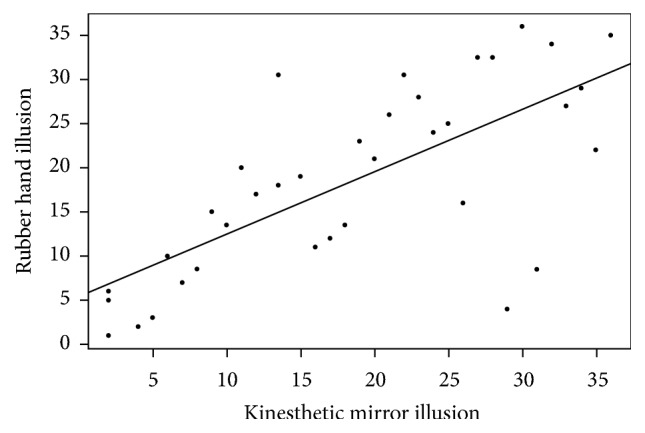
Relationship between the composite illusion strength index for the kinesthetic mirror illusion and the rubber hand illusion. The data were rank-transformed to appropriately illustrate the nonparametric analysis. The solid line represents the slope corresponding to the Spearman correlation coefficient.

As predicted, there was also a relationship between the strength of the kinesthetic mirror illusion and the strength of the rubber hand illusion. Measures of the same type were especially correlated across the two illusions, with all correlations greater than *ρ* = .60. In other words, participants experiencing larger proprioceptive drift for the kinesthetic mirror illusion also experienced greater proprioceptive drift for the rubber hand illusion, and the same was true for onset latency and subjective intensity. Importantly, measures of different types were also significantly correlated across the two illusions (with the exception of proprioceptive drift in the kinesthetic mirror illusion), indicating that the relationship was not due to similarities in measurement processes. The strong relationship between the strength of the two illusions was summarized by the significant correlation between the two composite illusion strength indices, *ρ* = .70, *p* < .001; this relationship is depicted in Figure 3. In summary, individual differences in sensitivity to the kinesthetic mirror illusion were related to individual differences in sensitivity to the rubber hand illusion: participants who responded strongly to one illusion tended to respond strongly to the other and vice versa.

To confirm that this relationship between indices was specific to a manipulation of the body schema, we tested whether responses in the illusion conditions were related to responses in the control condition of the rubber hand illusion (such analysis was not performed for the control condition of the kinesthetic mirror illusion since no illusion was reported). The composite index in the control condition of the rubber hand illusion was unrelated to the composite illusion strength index in the illusion condition of the rubber hand illusion (*ρ* = .11, *p* = .541) and in the illusion condition of the kinesthetic mirror illusion (*ρ* = .25, *p* = .135). In other words, the participants' responses were related to the two conditions eliciting a manipulation of the body schema, but they were not related to a condition involving no manipulation of the body schema.

### 3.4. Correlations between Sensitivity to Illusions and Traits Related to Eating Disorders

To test the hypothesis of a relationship between sensitivity to embodiment illusions and traits related to eating disorders, we assessed bivariate correlations between the two composite illusion indices, the BSQ and the EDI-II. All analyses were restricted to the 30 younger women in the sample (<22 years, excluding 6 participants with ages ranging from 28 to 46) to avoid confounding effects of age on the risk of developing eating disorders. The results are detailed in Table 3. Five correlations were significant, involving the subscales bulimia, ineffectiveness, and maturity fears. Replicating this analysis on the whole sample (*n* = 36) elicited even lower relationships between illusion strength and the questionnaires, with only two correlations remaining significant. In other words, the data suggested weak evidence of a relationship between body schema malleability and the risk of eating disorders, even in the younger participants.

## 4. Discussion

The main objective of the present study was to test the link between two bodily illusions tasks, supported by manipulation of sensory inputs and multimodal integration, namely, the rubber hand illusion task and the kinesthetic mirror illusion task. Of secondary interest, we aimed to test whether traits related to eating disorders in a subclinical population were linked to sensitivity to these illusions.

Participants demonstrated reliable individual differences in illusion sensitivity, as evidenced by the temporal stability of illusion strength across sessions and by the convergence between different measures of illusion strength within the same illusion task. As predicted, individual differences in sensitivity to the kinesthetic mirror illusion were also highly related to individual differences in sensitivity to the rubber hand illusion. These results are—to our knowledge—the first to evidence a relationship between the two illusions and support the possibility that they share a common mechanism, as suggested by several authors [14, 21, 25].

As mentioned in the introduction, there are several conceptual reasons to believe that this common mechanism is a transitory manipulation of the body schema. In the case of the rubber hand illusion, several arguments suggest that it is directly caused by modulation of the body schema. For example, the illusory feeling of touch is associated with activity in multiple sensory areas, including the ventral premotor cortex but also intraparietal cortices and the cerebellum [11]. As shown by [9], the activity in these areas reflects the integration of congruent multisensory signals from the body, rather than the visual representation of a single limb, suggesting that the illusion is indeed based on a modulation of the body schema as a whole. Although less research has been devoted to the underpinnings of the kinesthetic mirror illusion, it can also be interpreted in terms of body schema manipulation [14]. Our study provides support for this hypothesis by demonstrating a strong relationship with the rubber hand illusion.

At first glance, the effects of the mirror paradigm on kinesthesia (sense of movement), through manipulation of multisensory integration, may seem to be exclusively of visual origin [13], instead of involving modulation of other sensorial afferents such as muscular or tactile inputs. Indeed, visual feedback from reflecting limb appears to be a major factor in the effects of mirrors on kinesthesia [8], sense of position [40], but also reduction of pain [41, 42]. However, in the case of the kinesthetic mirror illusion, studies have shown that the illusory experience is produced by an interaction between visual afferences and other pieces of somatosensory information. The illusion reflects the integration between mirror visual signals and proprioceptive signals from the hidden hand [8, 20]. There is also an impact of volitional effort on the hidden hand: the occurrence and intensity of the kinesthetic mirror illusion are modulated by whether volitional effort on the limb behind the mirror is congruent or not with visual movement in the mirror [19]. A recent study highlighted that the kinesthetic mirror illusion can even survive to visual impoverishment, persisting despite visual covering of 84% or more of the mirror, whereas proprioceptive afferents from the arm facing the mirror are necessary to maintain the illusion of rapid displacement of the hidden arm [27]. These pieces of evidence indicate that the kinesthetic mirror illusion actually emerges from the integration of multisensory signals, reflecting construction and updating of the body schema as a whole [43]. As a consequence, the body schema hypothesis seems to provide the most explanatory power in the context of mirror paradigms in general and the kinesthetic mirror illusion in particular [14].

If both the rubber hand illusion and the kinesthetic mirror illusion can be interpreted in terms of body schema manipulation, individual differences in sensitivity to these illusions may be driven by individual differences in malleability of the body schema. In other words, sensitive participants would have a more malleable body schema when compared to nonsensitive participants. This hypothesis, first suggested to explain the extra sensitivity of patients with eating disorders to the rubber hand illusion [10], is compatible with our finding of individual differences that are both stable across sessions of trials and consistent across the two types of illusions. The importance of this result becomes even more obvious when one considers that we studied a nonclinical and homogeneous sample. Future research may be interested in generalizing this specific conclusion to other types of body schema illusions, such as the invisible body illusion [6] or the marble hand illusion [5]: indeed, the same pattern of individual differences should appear for any phenomenon based on the manipulation of the body schema, especially if they are induced based on the same sensory channels. Importantly, however, these individual differences may not generalize to all types of bodily illusions, as sensitivity to the kinesthetic mirror illusion does not seem related to sensitivity to other kinesthetic illusions induced based on proprioceptive inputs (see [8], for a study using mechanic vibration).

A secondary purpose of the present study was to test whether participants at risk of developing eating disorders would be more sensitive to both illusions. Indeed, one interesting consequence of the relationship between sensitivity to body schema illusions and eating disorders is that nonclinical participants with a malleable body schema might share common psychological traits with patients suffering from eating disorders. Sensitivity to embodiment illusions was unrelated to concerns about body shape as measured with the BSQ, but as predicted, significant correlations emerged with three subscales of the Eating Disorders Inventory-II:* bulimia*,* ineffectiveness*, and* maturity fears*. The* bulimia* subscale is directly related to eating disorders and represents the tendency of participants to engage in behaviors such as binge eating and self-induced vomiting. The relationship between bulimic behaviors and sensitivity to the rubber hand illusion is in line with the results of prior studies [29, 30], although the lack of a relationship between illusion sensitivity and the* drive for thinness* and* body dissatisfaction* subscales does not provide support for the hypothesis of a systematic link between illusion sensitivity and eating disorders. The* ineffectiveness* and* maturity fears* subscales reflect two psychological dimensions often associated with eating disorders; their relationship with illusion sensitivity is less straightforward to interpret but also hints at possible correlates between malleability of the body schema and psychological traits. Although these preliminary findings would obviously need to be replicated and extended in future studies, they suggest that sensitivity to embodiment illusions might constitute a marker of vulnerability to body misperception and ultimately to eating disorders, opening a promising line of research. This possibility would need to be explored in a larger sample and in participants with clinical eating disorders.

A final point of discussion concerns the relationships between the various measures of illusion strength. In the case of the rubber hand illusion, measures of proprioceptive drift (change in perceived position of the hidden hand) and subjective intensity (change in perceived limb ownership) are used interchangeably to measure illusion strength. However, some recent studies have shown dissociations between these two indices [44–46]. Whereas the subjective feeling of ownership does not occur in control conditions of the rubber hand, authors demonstrated that proprioceptive drift could occur in these conditions [46]. In another study, Nava and colleagues [45] used a particular form of nonvisual rubber hand illusion and showed that congenitally blind individuals did not demonstrate proprioceptive drift, whereas late blind and sighted individuals did. Yet all participants could feel the illusion. In a third study, Abdulkarim and Ehrsson [44] manipulated the position of the participants' hidden hand without them noticing. The authors showed that moving the participant's hand, either closer to or away from the rubber hand, did not change subjective intensity of the illusion. All three studies demonstrate that the subjective feeling of limb ownership in the rubber hand illusion does not vary exclusively as a function of perceived position of the real hand.

Echoes of these dissociations appeared in our own results. Overall, proprioceptive drift demonstrated the weakest correlations with other measures. In the case of the rubber hand illusion, proprioceptive drift was not significantly correlated with the other two measures of illusion strength, compatible with dissociations observed in the literature [44–46]. Taken together, these pieces of evidence suggest that the various indices of illusion strength may not fully reflect body schema manipulation to the same extent. One possibility is that proprioceptive drift constitutes an independent process that, under certain conditions, is linked with or caused by the subjective illusion of ownership. Nevertheless, our data still demonstrated strong correlations between all indices (even proprioceptive drift in the rubber hand illusion correlated .72 with proprioceptive drift in the kinesthetic mirror illusion), indicating that all measures—including proprioceptive drift—are adequate markers of illusion strength.

## 5. Conclusions

The present study is the first to show that there exist reliable individual differences in sensitivity to the kinesthetic mirror illusion and the rubber hand illusion and that participants sensitive to one illusion are also sensitive to the other. Overall, these results strongly support the idea that the two body schema illusions share the same mechanism. One possibility is that the two illusions represent a transitory modification of body schema, wherein the most sensitive people would have the most malleable body schema. Body schema illusions might constitute a marker of vulnerability to body malleability, to body misperception, and ultimately to eating disorders, opening a promising line of research.

## Supplementary Material

Raw data for both the kinesthetic mirror illusion and the rubber hand illusion.

## Figures and Tables

**Table 1:** Descriptive statistics for illusions strength measures.

Illusion	Parameters	First session			Second session		
		Med	MAD	Range	Med	MAD	Range
Kinesthetic mirror illusion	Proprioceptive drift (degrees)	10.63	10.63	0 to 67.19	13.13	13.13	0 to 63.13
	Onset latency (sec)	8.13	2.75	2.69 to 18	8.63	2.88	2.25 to 18
	Subjective speed (0 to 20)	5.63	2.13	0 to 10.5	6.13	2.88	0 to 10.38

Rubber hand illusion	Proprioceptive drift (cm)	1.25	1.25	−2.75 to 10.75	1.75	1.25	−0.13 to 8.63
	Onset latency (sec)	42.75	16.25	14.63 to 90	46.25	15.00	12.13 to 90
	Subjective intensity (1 to 10)	6.17	2.17	1 to 9.50	6.50	1.92	1 to 9.38

*Note.* Med = median; MAD = median absolute deviation (median of the absolute deviations from the median).

**Table 2:** Bivariate correlations between illusion strength measures.

Illusion	Measure	Kinesthetic mirror illusion				Rubber hand illusion			
		PD	OL	SS	CI	PD	OL	SI	CI
Kinesthetic mirror illusion	Proprioceptive drift	—							
	Onset latency	**−.38**	—						
	Subjective speed	**.58**	**−.69**	—					
	Composite index	**.81**	**−.79**	**.86**	—				

Rubber hand illusion	Proprioceptive drift	**.72**	**−.34**	**.39**	**.61**	—			
	Onset latency	−.27	**.60**	**−.57**	**−.50**	−.21	—		
	Subjective intensity	.25	**−.40**	**.61**	**.47**	.23	**−.51**	—	
	Composite index	**.55**	**−.59**	**.67**	**.70**	**.59**	**−.74**	**.80**	—

*Note*. PD = proprioceptive drift; OL = onset latency; SS = subjective speed; SI = subjective intensity; CI = composite index. Correlations were computed with Spearman's rho. Significant correlations are in bold.

**Table 3:** Bivariate correlations between illusion strength indices and eating disorders questionnaires.

Illusion	BSQ	EDI-II											
		Total	DT	Bu	BD	In	Pe	ID	IA	MF	As	IR	SI
KMI	.03	.16	.00	.30	.05	**.42**	.04	.07	.14	**.37**	.14	.23	.05
RHI	.03	.21	.02	**.39**	.06	**.43**	−.02	.22	.22	**.38**	.13	.28	.07

*Note*. KMI = kinesthetic mirror illusion; RHI = rubber hand illusion; DT = drive for thinness; Bu = bulimia; BD = body dissatisfaction; In = ineffectiveness; Pe = perfectionism; ID = interpersonal distrust; IA = interoceptive awareness; MF = maturity fears; As = asceticism; IR = impulse regulation; SI = social insecurity. Correlations were computed with Spearman's rho. Significant correlations are in bold.
